# Extra-pancreatic solid pseudopapillary neoplasm: a case report of primary testis origin and review of the literature

**DOI:** 10.3389/fonc.2025.1691249

**Published:** 2026-01-07

**Authors:** Qian Lu, Liming Yang, Shiyan Li

**Affiliations:** Department of Ultrasound in Medicine, Sir Run Run Shaw Hospital, Zhejiang University, School of Medicine, Hangzhou, China

**Keywords:** case report, extra-pancreatic solid pseudopapillary neoplasm, testicular mass, testis, β-catenin

## Abstract

Solid pseudopapillary neoplasm (SPN) is a rare tumor with low malignant potential, generally occurring in the pancreas. In this article, we report a case of extra-pancreatic solid pseudopapillary neoplasm of the testis (ESPN-T) of a 36-year-old male patient. The patient incidentally discovered a scrotal mass. Ultrasonography revealed a well-defined, regular cystic-solid lesion within the left testis. Magnetic resonance imaging (MRI) showed a nodular mass within the left testis, characterized by an isointense signal on T1-weighted imaging, a heterogeneous signal on T2-weighted imaging, and inhomogeneous enhancement after contrast administration. The patient subsequently underwent radical orchiectomy on the left side. The morphologic and immunohistochemical features of the testicular lesion confirmed the diagnosis of ESPN. Follow-up for 2 years after surgery revealed no signs of recurrence or metastasis. This is a rare case of SPN in the testis in China, which expands the differential diagnosis of testicular lesions and therapeutic strategies of ESPNs.

## Introduction

Solid pseudopapillary neoplasm (SPN) is an uncommon, low-grade malignant tumor of the pancreas, accounting for less than 3% of all pancreatic exocrine neoplasms (1–3). It predominantly affects young women and occurs more frequently in the head or tail of the pancreas (4–6). Since SPNs are generally asymptomatic or present with non-specific symptoms (7), most lesions are incidentally detected during imaging examinations. Extrapancreatic SPN (ESPN) is even rarer. To our knowledge, there are only a few reported cases of ESPNs within the testicles (8, 9), one of which occurred in the paratesticular position. More details about these seven cases are summarized in **Table 1**. Here we present one case of extra-pancreatic SPN of the testis (ESPN-T) in a young man in China. We summarize the imaging characteristics of this tumor, analyze it in combination with pathological features, and review previously published literature.

**Table 1:** Summary of solid pseudopapillary neoplasms of the testis or paratestis.

Case	Age (years)	Size (cm)	Localization	Histology and IHC
1	67	Diam 0.5	Testis (9)	Solid, fibrous septa of varying thickness IHC: positive for β-catenin (nuclear), CD10, vimentin, and NSE
2	38	Diam 2.1	Testis (9)	Solid, marked sclerotic background, occasional pseudopapillae IHC: positive for β-catenin (nuclear), CD10, focal NSE, and focal Syn
3	33	Diam 0.5	Paratestis (9)	Entirely cystic, no fibrosis IHC: positive for β-catenin (nuclear), CD10, CD56, vimentin, NSE, and Syn
4	24	3*3*2.5	Testis (9)	Solid, fibrous septa of varying thickness, occasional pseudopapillae IHC: positive for β-catenin (nuclear), CD10, focal CD56, vimentin, focal NSE, and focal Syn
5	82	2*2*2.5	Testis (9)	Partially cystic, fibrous septa of varying thickness, focal pseudopapillae IHC: positive for β-catenin (nuclear), CD10, focal CD56, focal NSE, and Syn
6	37	1*1*1.5	Testis (9)	Solid, fibrous septa of varying thickness IHC: positive for β-catenin (nuclear), CD10, focal CD56, vimentin, NSE, and Syn
7	47	Diam 3.0	Paratestis (8)	Solid, fibrous septa of varying thickness, papillary architecture IHC: positive for β-catenin (nuclear), CD10, CD56, vimentin, NSE, and Syn

## Case summary

**Figure 1:**
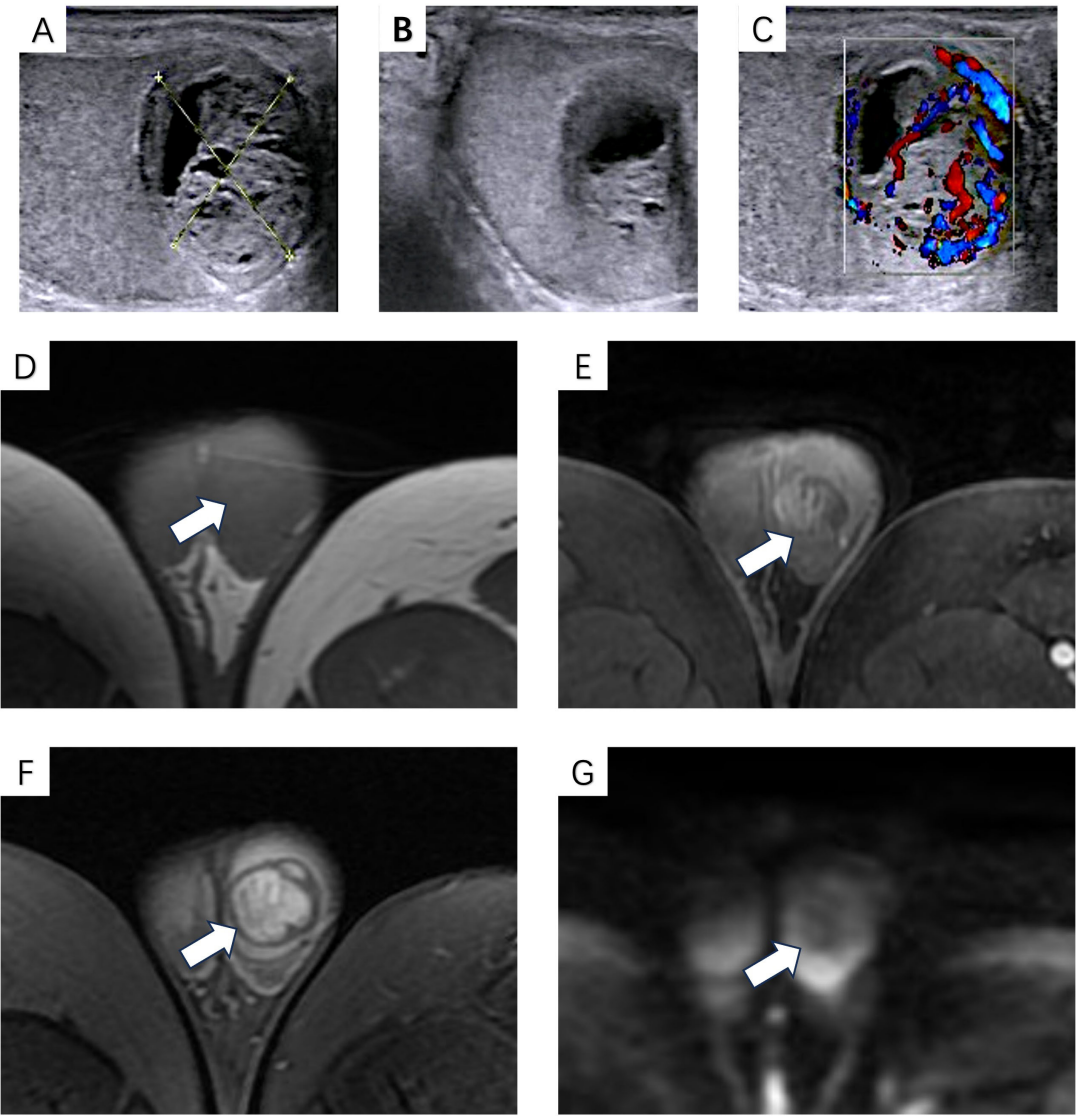
B-mode ultrasound showed a well-circumscribed, regular, solid-cystic mass located within the left testis; the solid component of the mass was predominant, and its rim is slightly hyperechoic **(A, B)**. Color Doppler imaging (CDFI) demonstrated peripheral and intratumoral blood flow signals **(C)**. MRI showed isointense signals on T1-weighted images **(D)** and heterogeneous signals on T2-weighted images **(E)**, with heterogeneous enhancement in the arterial phase **(F)**; no hyperintense signal was observed on DWI **(G)**.

A 36-year-old man noticed an indolent left testicular mass on self-palpation in 2021. Due to the absence of other symptoms, he did not seek medical attention and was followed for more than 2 years. At 1 week before his visit in 2023, he perceived an enlargement of the mass and visited our urology department. His physical exam revealed a 2-cm mass in the left testicle. The mass is moderately firm, with well-defined borders and moderate mobility. No previous medical or family history of genetic disorders was found. Subsequently, the scrotal grayscale ultrasound revealed a well-circumscribed, regular, solid-cystic mass located within the left testis. The solid component of the mass was predominant, and its rim was slightly hyperechoic. The largest section measured approximately 2 × 2 cm. Color Doppler imaging (CDFI) demonstrated the peripheral and intratumoral blood flow signals of the lesion (**Figure 1**).

For further diagnosis, the patient underwent a pelvic MRI examination. The contrast-enhanced pelvic MRI showed a 21 × 20-mm nodule within the left testis that was isointense on T1-weighted images and heterogeneous on T2-weighted images. After gadolinium administration, the lesion was enhanced heterogeneously. DWI shows no hyperintense signal in the lesion (**Figure 1**). Ultimately, the radiologist reports a germ cell tumor. Additionally, tumor markers such as CA-199, CA-125, AFP, CEA, and ferritin were all within normal ranges. The reproductive hormone levels were normal, except for a mild elevation in prolactin at 22.10 (normal range: 2.64–12.13 μg/L). Biochemical tests showed slight increases in cholesterol and uric acid. The complete blood count revealed no abnormalities.

**Figure 2:**
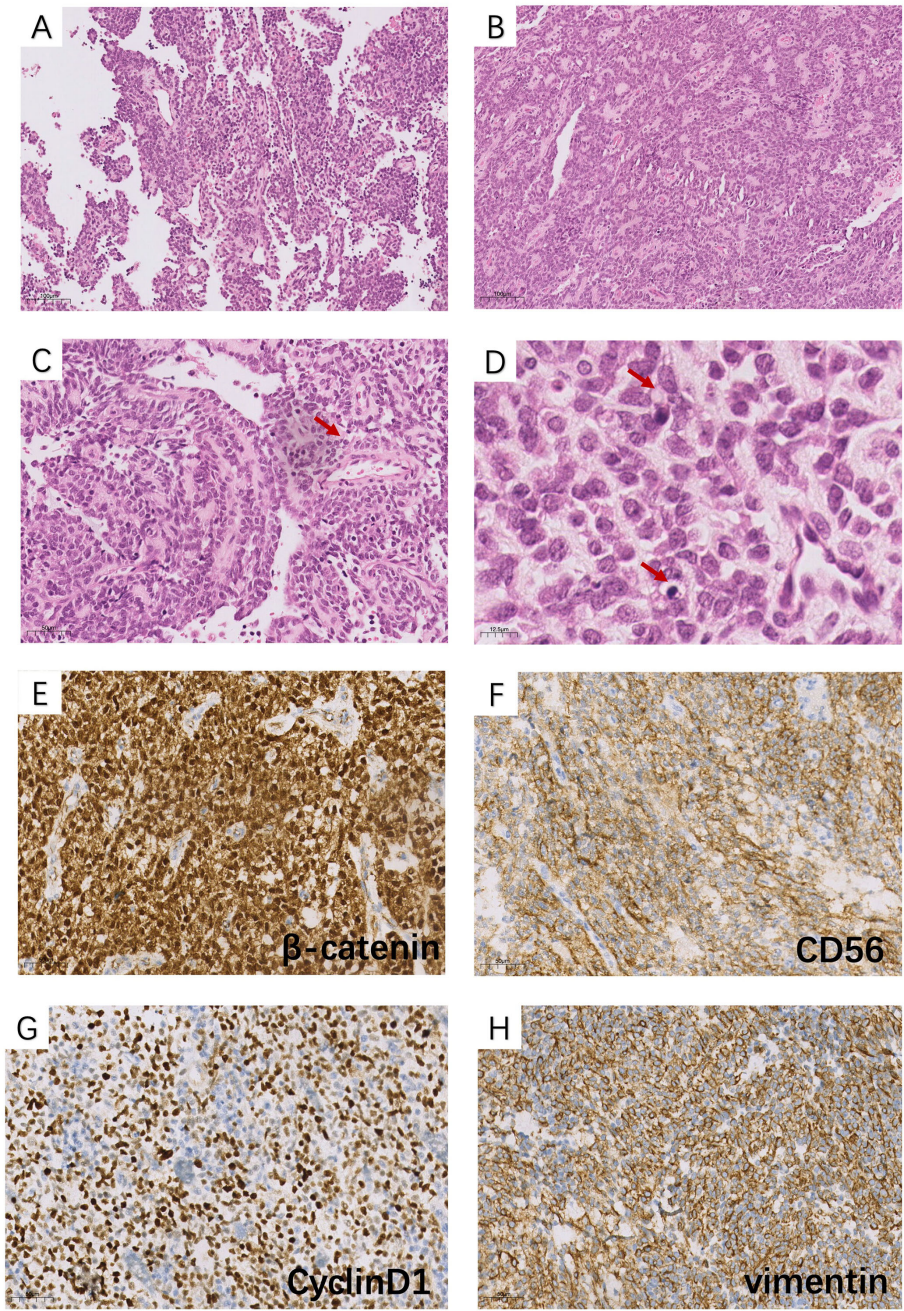
Histopathological features (H&E): **(A, C)** pseudopapillary structures (×100 and ×200 magnification); **(B)** solid nests and sheets of cells with an epithelioid appearance (×100 magnification); **(D)** in some foci, tumor cells had vacuolated cytoplasm, and some areas exhibit eosinophilic bodies (pointed to by the arrow) (×400 magnification). Immunohistochemical findings (×100 magnification): the tumor cells are positive for **(E)** β-catenin (nuclear and cytoplasmic), **(F)** cyclin D1, **(G)** CD56, and **(H)** vimentin.

**Figure 3:**
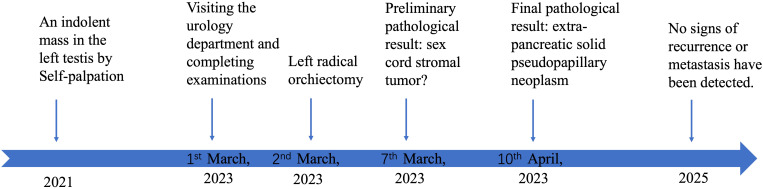
Treatment timeline diagram of the case.

Based on imaging and laboratory examinations, the patient was advised to undergo surgery. He underwent a left radical orchiectomy under general anesthesia. Macroscopically, the cross-section of the tumor appeared fleshy and fish-like in appearance. Histopathology showed that the neoplasm consists of solid sheets and nests of epithelioid cells arranged around vessels to form pseudopapillary structures. Delicate fibrous septa intermittently traversed the neoplastic tissue. The cells were monotonous, with visible nuclear grooves and rare mitoses. The tumor cells appeared with vacuolated cytoplasm in some places (**Figure 2**). Immunohistochemistry showed positive staining for nuclear β-catenin, partial CD56, vimentin, cyclin D1, and focal pan-CK (AE1/AE3). At the same time, the neoplastic cells exhibited negative results for CD10, HCG, AFP, inhibin, CD99, FLI-1, P504S, PLAP, OCT-4, CA117, Glypican-3, S-100, SALL4, FOXL2, SF-1, Syn, chromogranin A (CgA), and calretinin (**Figure 2**). After surgery, the patient received no additional treatment but was consistently followed up at the outpatient clinic. Follow-up examinations at the local hospital revealed no signs of recurrence, and the serum test showed no abnormalities. A treatment timeline diagram is shown in **Figure 3**.

## Discussion

The histogenesis of ESPNs remains elusive, with two main hypotheses proposed, namely: (1) origin from pancreatic progenitor cells with multidirectional differentiation capacity and (2) derivation from multipotent genital ridge cells that adhered to the pancreas during early embryogenesis. The latter is supported by the female predominance of SPNs, a relatively young age distribution, the lack of pancreatic markers, expression of sex hormone receptors, and occasional post-menopausal regression (10). Previous literature has revealed that ectopic pancreatic tissue has been recognized in organs like the mesentery, colon, and so on, which demonstrated that they shared embryologic derivation with the primitive duodenum (11–13). In the present case, however, no ectopic pancreatic tissue was identified, favoring the genital ridge hypothesis. Molecularly, most SPNs harbor mutations within exon 3 of the β-catenin gene (CTNNB1), thereby causing the nuclear accumulation of β-catenin and the activation of the Wnt/β-catenin pathway, which promotes tumorigenesis (14).

Compared to other neoplasms, identifying ESPNs based on imaging findings exhibits a slightly lower accuracy, but it still exerts an indispensable effect in the preliminary diagnosis of ESPNs. Among the most widely used imaging modalities, such as ultrasound, computed tomography (CT), and MRI, ESPNs share similarities with SPNs-P in imaging manifestations. At ultrasonography, SPNs-P typically are identified as round or oval lesions with heterogeneous hypoechogenicity, which may present as solid, cystic, or mixed morphology; some may show hypoechoic or anechoic fissures, while calcifications are relatively rare (15, 16). These tumors are often encapsulated and have a hyperechoic rim, with clear boundaries and regular shapes (17). CDFI commonly detects sparse blood flow signals due to the few and thin nutrient vessels of the tumor (16). Contrast-enhanced ultrasound (CEUS) exhibits marginal perfusion in the early arterial phase, and the interiors are heterogeneously enhanced (18). Under CT, the characteristic manifestation of SPNs is an encapsulated mass comprised of irregular solid and cystic components (19, 20). On MRI, lesions of SPNs usually exhibit a hypointense signal on T1-weighted images but may contain hyperintense signal foci due to internal bleeding; a heterogeneous high-signal may generally be seen on T2-weighted images (21), and diffusion-weighted imaging (DWI) presents a hyperintense signal (22). The solid part of the SPNs exhibited inhomogeneous enhancement in the arterial phase and progressive enhancement during the venous phase (23). However, these manifestations may vary with the ratio of solid/cystic components, presence of bleeding, or calcification (24). Moreover, MRI has the advantage in distinguishing tumors from surrounding normal tissues (2), which is somewhat significant in determining the degree of malignancy. In this case, grayscale ultrasound and MRI findings were analogous to those of SPNs, although CDFI in this case showed peripheral and internal blood flow signals. Inconsistent results may be explained in future analyses with larger sample sizes. Unfortunately, due to various limitations, CEUS was not performed for auxiliary diagnosis.

A definitive diagnosis of ESPNs relies on pathology ultimately. Microscopically, the tumor consists of morphologically uniform, poorly adherent epithelioid tumor cells arranged in solid nests as well as reticular and trabecular patterns. One or more layers of tumor cells encircle the fibrovascular axis, forming pseudopapillary projections (25). In addition to these typical characteristics, the tumor cells in some lesions have vacuolar cytoplasm (20). Eosinophilic granules may be visible within or outside the cytoplasm (26). Generally, immunohistochemical staining should include β-catenin, CD10, CD56, vimentin, E-cadherin, CgA, cytokeratin (CK), and synaptophysin (Syn) (27, 28) or at least include the first five. Immunohistochemical staining for markers such as CD10, vimentin, CD56, and cyclin D1 in tumors is generally positive, particularly for β-catenin (10). CK and Syn may show varying degrees of expression. CgA, inhibin, or E-cadherin is mostly negative (15). The diffuse expression of β-catenin in cells is considered an essential indicator of pathogenesis, linked to the activation of the Wnt/β-catenin pathway (29). Extra-immunohistochemical markers required for ESPNs should be determined based on the specific organ involved (25, 30). In our case, all significant markers associated with germ cells (SALL4, AFP, HCG, OCT3/4, and Glypican-3) and sex cord-stromal cells (inhibin, SF-1, and S-100) were negative. This supports the conclusion that the tumor in this case did not originate from either of these sources. Unexpectedly, β-catenin, CD56, vimentin, and cyclin D1 stainings were positive in the case. Based on morphologic features and IHC, the present case was confirmed to have the diagnosis of ESPN. Compared to some previous cases, our tumor exhibits slight differences, as it is negative for CD10 and Syn. Moreover, we did not include all associated immunohistochemical markers in our testing, considering the financial burden on patients and their non-critical role in diagnosis.

As a mass within the testicle, germ cell tumors (GCTs) are the primary consideration in differential diagnosis, with mixed germ cell tumors (MGCTs) being the second most common subtype after seminomas (31). TMGCTs exhibit similar imaging features to our case, presenting as cystic-solid masses (31–34). Clinical manifestations and laboratory test discrepancies hold greater initial differential diagnostic value. TMGCTs are aggressive malignancies that grow rapidly, causing scrotal swelling and early metastasis (particularly when accompanied by highly invasive subtypes), which may subsequently lead to metastasis-related symptoms (35). Additionally, some TMGCTs may be associated with elevated alpha-fetoprotein or human chorionic gonadotropin levels (36). More detailed descriptions are presented in **Table 2**. Furthermore, previous reports have described a rare tumor arising in the testis, designated as the “primary testicular signet-ring stromal tumor” (PTSRST) (37). This entity, which has 13 reported cases, also expresses β-catenin and is morphologically similar to SPN to a certain extent, but the former also contains a signet ring cell component in microscopy (9, 38). In subsequent studies, the authors hypothesized that the two belonged to the same category. The hypothesis posits that signet ring cell components are present in the initial stage, and as the tumor expands, solid pseudopapillary components become more ascendant. However, subsequent studies have challenged this notion: detailed comparisons reveal statistically significant differences in morphology and immunohistochemical profiles (39), and PTSRSTs are generally smaller than ESPN-T, leading to the conclusion that a blanket classification would be premature. In addition, there is also a difference in size between the two entities, with PTSRST being smaller than ESPN-T (9, 22). To avoid misclassification, the diagnostic workup should include a careful morphological assessment and an extended immunohistochemical panel to exclude sex-cord stromal differentiation (40). Attention to subtle cytologic details, the inclusion of sex cord stromal and germ cell markers, and consideration of tumor size may assist in differentiation; however, further larger-scale sample studies are needed to validate these clues.

**Table 2:** Differential diagnosis of our case.

Feature category	Our case	TMGCTs (34, 48)	PTSRSTs (8, 38)
Imaging	US: a well-circumscribed, regular, solid-cystic mass with a hyperechoic rim MRI: isointense signal on T1-weighted imaging, heterogeneous signal on T2-weighted imaging, and inhomogeneous enhancement after contrast administration	US: poorly defined heterogeneous masses with areas of necrosis, hemorrhage, fibrosis, cystic changes, and calcifications MRI: heterogeneous areas on T1- and T2-weighted MRI that are due to cystic change, hemorrhage, or necrosis	US: heterogeneous hypoechoic round lesion
Clinical manifestations	Asymptomatic	Scrotal swelling; related symptoms if metastasized	Not described
Laboratory examinations	Normal serum tumor marker levels	Elevated serum AFP or HCG levels (non-universal)	Normal serum tumor marker levels
Histopathology	Solid sheets and nests of epithelioid cells are arranged around vessels to form pseudopapillary structures. Delicate fibrous septa intermittently traversed the neoplastic tissue	Anaplastic-like cells exhibiting diverse architectural patterns; some may be accompanied by hemorrhage, necrosis, and vascular infiltration	The pattern is monomorphic, consisting of circular epithelial-like cells forming clusters of signet-ring cells containing a single large cytoplasmic vacuole. These signet-ring cells are separated by fibrous septa into beam-like and nests-like structures. Some areas may lack signet-ring cells but are instead composed of cells with eosinophilic cytoplasm
IHC	(+): β-catenin, vimentin, and cyclin D1; Partial (+): CD56 and pan-CK; (-): CD10, SALL4, SF-1, PLAP, OCT-4, HCG, AFP, inhibin, CD99, FLI-1, P504S, CD117, CD30, Glypican-3, S-100, FOXL2, Syn, CgA, and CR	(+): SALL4, PLAP, OCT3/4, glypican-3, α-fetoprotein, β-hCG, and GATA3; (-): β-catenin, Syn, CgA, and inhibin	(+): β-catenin, CD10, vimentin, CD56, NSE, and cyclin D1; (-): SALL4, SF-1, OCT-3/4, inhibin, CR, S-100, and CgA

(+), positive; Partial (+), partial positive; (-), negative.

Reviewing previous studies, we found that surgery was the primary treatment for ESPN (22, 41–44). In most cases, no subsequent therapy was recommended post-surgery, with only follow-up visits advised. Currently, the sample size for ESPN is limited, and its treatment and follow-up strategies essentially reference those of SPN. Only a very small number of highly aggressive tumors experienced rapid recurrence after surgery, leading to subsequent chemotherapy with poor prognosis (28). However, there is currently no consensus on specific chemotherapy regimens (2). The recommended chemotherapy regimens include gemcitabine, FOLFOX, FOLFIRI, ifosfamide, cisplatin, and etoposide (45). ESPNs, like SPNs, are considered as indolent tumors, and patients undergoing complete resection generally have favorable outcomes. The risk factors for recurrence include incomplete resection, large tumor size, younger patient age, tumor rupture, and male gender (46, 47). For high-risk patients, follow-up should be intensified.

In conclusion, this case expands the imaging, clinical, and morphological spectrum of ESPNs by documenting a rare primary testicular origin (ESPN-T). ESPN-T exhibits indolent biological behavior, as evidenced by the 2-year post-surgical follow-up, with no recurrence or metastasis. Complete surgical excision remains the cornerstone of treatment. While ESPNs are generally considered low-malignant-potential neoplasms, isolated cases (particularly in men) have shown metastasis and disease-related mortality, emphasizing the need for long-term surveillance. Further studies with larger sample sizes are warranted to clarify the classification of such lesions and refine diagnostic and management strategies.

## Data Availability

The original contributions presented in the study are included in the article/supplementary material. Further inquiries can be directed to the corresponding author.
